# Technologies to Support Frailty, Disability, and Rare Diseases: Towards a Monitoring Experience during the COVID-19 Pandemic Emergency

**DOI:** 10.3390/healthcare10020235

**Published:** 2022-01-26

**Authors:** Daniele Giansanti, Antonia Pirrera, Paola Meli, Mauro Grigioni, Marta De Santis, Domenica Taruscio

**Affiliations:** 1 Centro Nazionale Tecnologie Innovative in Sanità Pubblica, Istituto Superiore di Sanità, 00161 Rome, Italy; antonia.pirrera@iss.it (A.P.); paola.meli@iss.it (P.M.); mauro.grigioni@iss.it (M.G.); 2 Centro Nazionale Malattie Rare, Istituto Superiore di Sanità, 00161 Rome, Italy; marta.desantis@iss.it (M.D.S.); domenica.taruscio@iss.it (D.T.)

**Keywords:** COVID-19, SARS-CoV-2, frail people, rare diseases, remote assistance, remote rehabilitation, survey, technology

## Abstract

This report illustrates the design and results of an activity of surveillance proposed by the National Centre for Innovative Technologies in Public Health and the National Centre for Rare Diseases of the *Istituto Superiore di Sanità* with the aim of monitoring the state-of-use of technologies by people with frailty, disabilities, and rare diseases. The results of the surveillance activity reported in this report are as follows: *(a)* *An international Webinar*; *(b)* *A Full report published by the Istituto Superiore di Sanità* (ISS); *(c)* an electronic survey tool, for periodic monitoring; *(d)* an initial summary of the survey (15 September–30 November 2020), giving an overall picture relating to the state-of-use of technologies by the interviewed; *(e)* an understanding of the needs that emerged, causing reflection on the current state-of-the-art and offering important stimuli for all the stakeholders involved.

## 1. Introduction

The World Health Organization estimates that over one billion people live with some form of disability [1]. This corresponds to approximately 15% of the world population, with up to 190 million (3.8%) people aged 15 and over. The number of people with disabilities is also increasing, due to the progressive aging of the population and the increase in chronic health conditions. Disability is extremely varied, and some associated clinical situations can result in pathological conditions that require extensive healthcare needs. However, in general, all people with disabilities, as well as all other citizens, have the right to access traditional health services. Although Article 25 of the United Nations Convention on the Rights of Persons with Disabilities reinforces the rights of people with disabilities to achieve the highest standards of healthcare without discrimination, in reality, there are still few countries that provide adequate and quality services.

Furthermore, very few countries collect disaggregated data by disability in the health sector, and this has become much more evident and burdensome during the emergency caused by COVID-19; there has been no consistent inclusion in the responses put in place to control the pandemic. People with disabilities do not always receive adequate support. On the contrary, they are often exposed to risks with serious consequences of contracting COVID-19, develop severe COVID-19 symptoms, and have the potential of worsening health, both during and after the pandemic [2].

Focusing attention on the national territory, Istat (The Italian National Institute of Statistics) estimates that 3.1 million disabled people, in Italy, constitute 5.2% of the resident population [3]. Of these, almost 1.5 million are represented by the elderly over 75 (i.e., more than 20% of the population in that age group). If we also add to this number the people who declare they have minor limitations, the total number of people with disabilities in Italy rises to 12.8 million. There is talk of different types of disabilities, ranging from the highest degree of difficulty in the essential functions of daily life, to much milder limitations, including chronic diseases such as diabetes, heart disease, chronic bronchitis, liver cirrhosis or malignancy, senile dementias, behavioral disorders, and rare diseases [3,4].

It is evident that, for such a large group of citizens with specific needs and fragility, technological resources represent an indispensable tool for the continuity of care/therapy, and, in the COVID-19 era, these are transformed into an essential lifeline. We should consider that there have been several new technological proposals because of COVID-19, such as [5,6], but not with a special focus on frail people. Accessibility and the use of technologies are not only a current issue; they are also vital for persons living with a disability, because they can make a significant difference to life quality. This depends a great deal on both the offer of continuity of remote care and on how it is possible to cope with the problem of the *Digital Divide* that, where available, hinders access. The *Digital Divide* concerns the gap between those who have effective access to information technologies and those who are partially or totally excluded from it. The *Digital Divide* has three polarities/levels of intervention. The *first level* of the *Digital Divide* is represented by the difficulty in access to the infrastructures; this remains a problem, even in the richest and most technologically advanced countries in the world [7]. The *second level* is represented by literacy, characterized by the skills that enable individuals to seek, understand, and use information in ways that promote and maintain health based on Digital Health [8]. The *third level* is represented by the potential benefit level [9]. This concerns the extent to which economic, cultural, social, and personal types of engagement with the Internet result in a variety of economic, cultural, social, and personal outcomes. The three levels of the digital divide are also evident during the pandemic [10,11,12,13,14,15], where digital resources are fundamental.

## 2. The Idea of the Surveillance Project

The COVID-19 pandemic and the consequent obligation of social distancing has offered a great stimulus for the development of digital technologies for the continuity of treatments and cures; however, the limits of effective access to these digital technologies have often exacerbated the disparity [15], accentuating the difficulties that “frail people”, their families, and caregivers face daily.

The *National Center for Innovative Technologies in Public Health* (Centro Nazionale Tecnologie Innovative in Sanità Pubblica, TIPS), together with the *National Center for Rare Diseases* (Centro Nazionale Malattie Rare, CNMR), with the collaboration of the Press Office of the Istituto Superiore di Sanità (ISS) and internal and external experts of the ISS, has developed an online survey entitled “Technologies to support frailty, disability and rare diseases: the COVID-19 experience”.

The study had several objectives.

The *first objective* was to design the survey electronically in order to easily administer it, by submitting it and collecting data easily using mobile technology.

The *second objective* was to identify which technologies were used during home isolation and physical and social distancing, to carry out, where possible, daily activities (work, school, etc.) and health and social-health treatments in a period in which all facilities and services have been closed or suspended.

The *third objective* was to monitor and identify the real accessibility and usability of the technologies currently available by “frail people”, their families, and caregivers.

The *fourth objective* was to disseminate the results orally by means of a Webinar to an international audience in order to compare and discuss solutions.

The *fifth objective* was to disseminate nationally and internationally the results in a way that is useful for both stakeholders and citizens.

## 3. Methods

The online electronic survey, *The Central Tool*, reaches its target subjects through the most common web communication tools (e-mail, social media, etc.) by simply sending a link that allows direct access to the survey and provides preliminary results in real time. Furthermore, in the specific case of the COVID-19 emergency context, the online survey was also able to overcome the restrictions of social distancing.

In this study, Microsoft Forms was chosen, which is available in the Office 365 suite provided to the staff of the Istituto Superiore di Sanità and which, for this reason, respects the IT security aspects required by current regulations from a systems point of view. The following modules were used:

- Single choice questions;
- Multiple choice questions;
- Evaluation (graded) questions with a 6-level psychometric scale;
- Likert questions [16] with a 6-level psychometric scale;
- Open-ended questions (in a few cases).

The dissemination took place through the web pages of the ISS site, the thematic site of the Ministry of Health (www.malattierare.gov.it, accessed on 23 January 2021) [17], and Uniamo—the Federation of rare diseases (www.uniamo.org, accessed on 23 January 2021) [18]. Furthermore, news was provided via the ISS Rare Diseases Toll-Free Telephone line, the sites of reference associations such as the Interregional Working Group for Electronic and IT aids for the disabled (GLIC), and the Scientific Association for Digital Health, as well as by social media such as the Facebook, Linkedin, Twitter, and Instagram accounts of various entities and institutions.

To minimize the potential bias caused by the *Digital Divide*, we invited (during submission) those more familiar with technology to support the less familiar.

As regards the questions of type (c) with 6-level evaluation and the Likert questions in (d) (e.g., Question 23) with sub-questions at 6 levels, it was possible to assign a minimum score of 1 and a maximum of 6; therefore, the value theoretical mean (TM) is 3.5. This value can be referred to by comparison in the analysis of the answers. An average response value below TM indicates a more negative than positive response. An average value above TM indicates a more positive than negative response.

## 4. Results and Discussion

The study, in line with the objectives, produced several important results.

**Figure 1:**
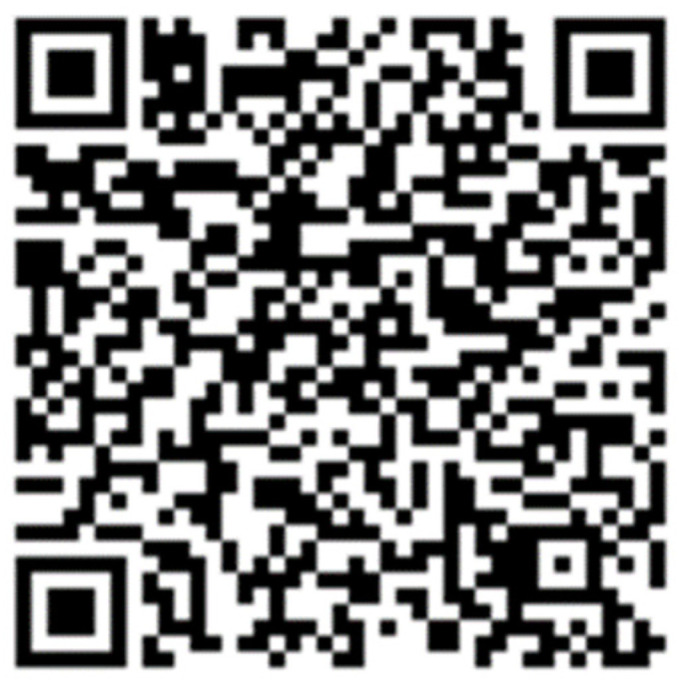
The QR Code.

The *first result* is the survey [19], accessible via:

- Internet link representing a mirror version, identical (with all ramifications) to the submitted copy (now closed and no longer reachable): [19].
- Quick Response Code (QR Code) (Figure 1); for those with only the paper version of the document, there is a Quick Response Reader available on most smartphones.

The *second and the third results* are the two disseminative products of the study:

- The international oral dissemination was carried out through a Webinar, using the resources of the *Istituto Superiore di Sanità*, the Italian National Institute of Health (NIH), coordinated by the National Centre for Rare Diseases. This Webinar was the 17th online webinar meeting organized by the National Centre for Rare Diseases. It was titled “17° Scientific Meeting Online COVID-19 and Rare Diseases, 28 January 2021 (h 15.00–16.30 CET), Istituto Superiore di Sanità (Rome), Italy Aula Pocchiari, and it involved several groups in the discussion [20].
- The outcome dissemination was carried out by means of the publication of Rapporti ISS COVID-19 (COVID-19 Reports). The COVID-19 Reports are aimed at healthcare professionals to address the different aspects of the pandemic. They provide essential and urgent information for emergency management and are subject to updates. They are produced by the COVID ISS working groups, made up of researchers from the *Istituto Superiore di Sanità*, who can also work in collaboration with other institutions. We published it in English [21] to allow the scientific community who had followed the Webinar to directly read the outcomes in an extensive manner.

The *fourth result* is the summary of the current state that the responses received could take, highlighting the main problems encountered by fragile citizens and their families. It emerged from the 313 frail people interviewed in the pandemic period:

- There has been an increase in the use of generic *eHealth* and *mHealth* technologies and communication and messaging tools, which all represent an *essential lifeline* [10,11].
- There was a general difficulty in using and/or accessing specialized technologies for treatment or rehabilitation, with insufficient remote support for continuity of care.
- There was a strong desire to be able to access and use technologies appropriately, also through specific training that allows them to exploit their full potential.

The *fifth result* is that the data collected has revealed important critical issues that should be acknowledged by bodies and institutions.

The sample of 313 frail people had a normal distribution in relation to age. We tested the normal distribution of age by the Smirnov–Kolmogorov test of normality, which is suitable for samples such as ours [22]. The null hypothesis was that our data follows a normal distribution. We achieved *p* = 0.51. Because *p* > 0.05, we accepted the null hypothesis. We are therefore faced with a normal distribution.

Considering the enormous global upheaval, with drastic and sudden closures of social and health facilities due to the pandemic, the following is apparent:

- Only *9.2*% of respondents (29 persons out of 313 people) had benefited from remote rehabilitation and/or therapeutic support technologies;
- Of these, 31% (of this subsample of 9.2%, i.e., 9 out of 29 people) encountered problems and difficulties in using the tool effectively.
- Over *90*% of fragile subjects (282 out of 313 people) who participated in the questionnaire believed that the technology could be useful during the pandemic and in the future.

We applied a frequency test to estimate significance (χ^2^) [23].

The first *χ*^2^ test was applied to the first group of 9.2% (29 out of 313 people) who had benefited from technology. The tested hypothesis was the significance of the difference in frequency between the group of 29 people who accessed the services and the group of 284 people who did not access it.

The χ^2^ test returned a highly significant outcome, as Equation (1) shows:

(1) $$\chi 2=\frac{{\left(284-156.5\right)}^{2}}{156.5}+\frac{{\left(29-156.5\right)}^{2}}{156.5}=207.7\;\;p<<0.005$$

The second χ^2^ test was applied to 31% of the above subgroup of 29 people who accessed the technologies (9 out 29 people) but encountered problems in the use of the technology. The tested hypothesis was the significance of the difference in frequency between the group of 9 people who accessed the services and encountered problems and the group of 20 people who accessed the services without problems. The χ^2^ test returned a significant outcome, as Equation (2) shows:

(2) $$\chi 2=\frac{{\left(9-14.5\right)}^{2}}{14.5}+\frac{{\left(20-14.5\right)}^{2}}{14.5}=4.2,\;\;p<0.05$$

This result is very important, especially if we consider the fact that only 20 people (29 people accessing the technology minus 9 people with difficulties during the use of the technology) among the total of 313 accessed the technology in a satisfactory way.

The third χ^2^ test applied to over 90% of people (282 out of 313) who believed that “the technology could be useful during the pandemic and in the future”. The tested hypothesis was the significance of the difference in frequency between the group of 282 people who believe that “the technology could be useful during the pandemic and in the future” and the group of 31 people who had the opposite opinion. The χ^2^ test returned a highly significant outcome, as Equation (3) shows:

(3) $$\chi 2=\frac{{\left(282-156.5\right)}^{2}}{156.5}+\frac{{\left(31-156.5\right)}^{2}}{156.5}=201.3,\;\;p<<0.005$$

These significant results, in synthesis, made it possible to verify that: (a) very few people accessed, in a satisfactory manner, the technology; (b) a concrete and accessible solution was not found for the clear demand/expectation of technology, and (c) in consideration of this, it would be important to investigate the causes in order to propose effective interventions that also consider the tools suggested by the interviewees. These results also highlight the urgent need to implement innovative technological platforms and tools, but also to provide training courses for professionals, frail people, and their family members/caregivers and ultimately support services that offer constant assistance and also, where needed, psychological support for families.

## 5. Conclusions and Future Work

This report illustrates the design and results of an activity of surveillance proposed by the National Centre for Innovative Technologies in Public Health and the National Centre for Rare Diseases of the Istituto Superiore di Sanità, with the aim of investigating the state-of-use of technologies by people with frailty, disabilities, and rare diseases. The core element was an online questionnaire that was developed with simple and effective electronic tools based on Microsoft Forms, made available to ISS users, which was used during the COVID-19 pandemic, but would also be useful with simple upgrades in other periods. The project allowed the provision of a first overall summary relating to the state-of-use of technologies by citizens with fragility, disabilities, and rare diseases. From the summary emerged both the needs of the interviewees and the potential of the technology, causing reflection on the current state-of-the-art and offering important stimuli for the stakeholders involved. In fact, the demand for the supply of technology for continuity of care was not matched by an adequate supply response. The project also highlighted the usefulness of the disseminative tools set up by the Istituto Superiore di Sanità. Based on the evidence reported, some actions are planned for the future. In particular, it is deemed necessary to promote specific initiatives, some of which may be proposed directly by the TISP Centre and the CNMR Centre, jointly with all the other stakeholders involved in these issues. Among these could be, for example, awareness campaigns and training courses, or the elaboration of recommendations and documents with good practices useful to other competent bodies at national and international levels. Furthermore, it is considered to be of primary importance that this survey is used for periodic monitoring of these issues, both during and after the pandemic period, in order to plan appropriate strategies tailored to frail people. The authors and the two Centers, after this positive collaboration experience, are continuing the collaboration on surveillance activities, in a larger group in a project in collaboration with other national and international bodies, such as the WHO and the CENSIS (an Italian national socio-economic research institute for surveys to the Italian people), dealing with the assistive technologies on the population.

## References

[1] World Health Organization (2020). Disability and Health. Key Facts.

[2] Disability Considerations during the COVID-19 Outbreak. https://www.who.int/publications/i/item/WHO-2019-nCoV-Disability-2020-1.

[3] Istituto Nazionale di Statistica (2019). Conoscere Il Mondo della Disabilità: Persone, Relazioni e Istituzioni.

[4] Paolini M.C. I Numeri della Disabilità in Italia. Le Nius 20/3/2020. https://www.lenius.it/disabilita-in-italia/#:~:text=Secondo%20Istat%20sono%203%2C1,%2C2%25%20della%20popolazione%20italiana.&text=Se%20a%20questo%20numero%20aggiungiamo,sale%20a%2012%2C8%20milioni.

[5] Ramallo-González A.P., González-Vidal A., Skarmeta A.F. (2021). CIoTVID: Towards an Open IoT-Platform for Infective Pandemic Diseases such as COVID-19. Sensors.

[6] Nasr M., Islam M.M., Shehata S., Karray F., Quintana Y. (2021). Smart Healthcare in the Age of AI: Recent Advances, Challenges, and Future Prospects. IEEE Access.

[7] van Deursen A.J., van Dijk J.A. (2019). The first-level digital divide shifts from inequalities in physical access to inequalities in material access. New Media Soc..

[8] Neter E., Brainin E., Baron-Epel O. (2021). Group differences in health literacy are ameliorated in ehealth literacy. Health Psychol. Behav. Med..

[9] Van Deursen A.J., Helsper E.J. (2018). Collateral benefits of Internet use: Explaining the diverse outcomes of engaging with the Internet. New Media Soc..

[10] Gabbiadini A., Baldissarri C., Durante F., Valtorta R.R., De Rosa M., Gallucci M. (2020). Together Apart: The Mitigating Role of Digital Communication Technologies on Negative Affect During the COVID-19 Outbreak in Italy. Front. Psychol..

[11] Shah S.G.S., Nogueras D., Van Woerden H.C., Kiparoglou V. (2020). The COVID-19 Pandemic—A pandemic of lockdown loneliness and the role of digital technology: A viewpoint (Preprint). J. Med. Internet Res..

[12] Kondylakis H., Katehakis D.G., Kouroubali A., Logothetidis F., Triantafyllidis A., Kalamaras I., Votis K., Tzovaras D. (2020). COVID-19 Mobile Apps: A Systematic Review of the Literature. J. Med. Internet Res..

[13] Lai J., Widmar N.O. (2020). Revisiting the Digital Divide in the COVID-19 Era. Appl. Econ. Perspect. Policy.

[14] Shek D.T.L. (2021). COVID-19 and Quality of Life: Twelve Reflections. Appl. Res. Qual. Life.

[15] Bakhtiar M., Elbuluk N., Lipoff J.B. (2020). The digital divide: How COVID-19′s telemedicine expansion could exacerbate disparities. J. Am. Acad. Dermatol..

[16] Survey Monkey Cos’è una scala Likert?. https://it.surveymonkey.com/mp/likert-scale/.

[17] Insieme nel Mondo Delle Malattie Rare. Ministero della Salute, Istituto Superiore di Sanità. www.malattierare.gov.it.

[18] Web of Uniamo. www.uniamo.

[19] Technologies to Support Disabilities, Frailties and Rare Diseases: The COVID-19 Experience (Electronic Questionnaire). https://forms.office.com/Pages/ResponsePage.aspx?id=DQSIkWdsW0yxEjajBLZtrQAAAAAAAAAAAAZAAOUXdFhUNjkwQ0ZVRjhVWlVQVVVNTU1SSTlNTklZOC4u.

[20] 17° Scientific Meeting Online COVID-19 and Rare Diseases. https://drive.google.com/file/d/1f5_HkFecZ5Ck_Sw-5L7Pmfll8GvGx_Mz/view?usp=sharing.

[21] Giansanti D., Pirrera A., Renzoni A., Meli P., Grigioni M., De Santis M., Taruscio D. (2021). Technologies to Support Frailty, Disability and Rare Diseases: Development and Submission of a Survey during the Pandemic Emergency COVID-19. Version of June 18, 2021.

[22] (2008). Kolmogorov–Smirnov Test. The Concise Encyclopedia of Statistics.

[23] Balakrishnan Vassilly Voinov N., Nikulin M.S. (2013). Chi-Squared Goodness of Fit Tests with Applications.

